# Editorial: Highlights in abdominal and pelvic pain 2021/2022

**DOI:** 10.3389/fpain.2023.1097072

**Published:** 2023-02-16

**Authors:** Xiao-Ping Wang, Bo Wang

**Affiliations:** ^1^ Jiading Branch of Shanghai General Hospital, Shanghai Jiao-Tong University School of Medicine, Shanghai, China; ^2^ Zhongshan Hospital, Fudan University, Shanghai, China

**Keywords:** neuropathy, abdominal and pelvic pain, irritable bowel syndrome, acupuncture, VSL#3, endometriosis

**Editorial on the Research Topic** Highlights in abdominal and pelvic pain 2021/2022

It is our great pleasure to organize this Research Topic in Frontiers in Pain Research: *Highlights in Abdominal and Pelvic Pain 2021/22*. This Research Topic has a selection of high-impact articles authored by four groups of excellent international leaders in the field of pain research and has shared varied theories and/or experiences in the clinical treatment and therapy of abdominal and pelvic pain research. The work presented here highlights the broad diversity of research carried out in the abdominal and pelvic pain section, not specifically limited to years, and aims to highlight the main areas of interest among many clinical departments: anesthesiology and pain medicine, neurology, obstetrics and gynecology, urology, gastroenterology, immunology and allergy, surgery and orthopedics, and general medicine. Of course, state-of-the-art concepts, such as the brain-gut axis, or vice versa, also exist in some research collection papers (1, 2).

The first review article, *Importance of Non-pharmacological Approaches for Treating Irritable Bowel Syndrome: Mechanisms and Clinical Relevance* by Orock et al., introduced the updated situation on chronic visceral pain that represents an important unmet clinical need, with the severity of pain preventing individuals from participating in day-to-day activities and negatively affecting their quality of life. Although chronic visceral pain can be multifactorial, with many different biological and psychological systems that contribute to the onset and severity of symptoms, the main trigger is exposure to emotional and physical irritation, especially gastrointestinal disorders (GI) such as irritable bowel syndrome (IBS). To date, pharmacological treatments for patients with chronic visceral pain generally lack efficacy and are fraught with unwanted side effects. Cognitive behavioral therapy has emerged as a psychotherapy that shows efficacy in ameliorating stress-induced chronic visceral pain. It is interesting to read these articles on acupuncture mechanism disputes for disorders of the digestive system and the potential cellular mechanism (3, 4). Clinical acupuncture trials on the efficacy of treating symptoms of IBS have not yet reached a consensus; however, clinical efforts have been confirmed to reduce pain perception in patients. Experimental studies in rats identified that neurogenic inflammatory spots corresponded to acupuncture points in patients.

The second article, a wonderfully novel article about probiotic organisms identified in stool that were significantly correlated with improvement in colonic permeability and clinical symptoms, led future studies to investigate the mechanistic roles of VSL#3 and colonic permeability in the pathophysiology of IBS in a larger randomized controlled trial (submitted by Boonma et al.). It is well known that IBS is more common in children than in adults and that it affects the quality of human life. Once considered a disorder caused by abnormalities in gut smooth muscle, visceral hypersensitivity, and/or central nervous system hypervigilance, a change in focus has been observed to include both central and peripheral mechanisms in pathophysiology in the past decade. Related peripheral mechanisms include abnormal colonic transit and rectal evacuation, intraluminal intestinal irritants, altered gut microbiome, and enteroendocrine cell dysfunction. The United States Food and Drug Administration approved the authors to conduct a thorough safety study of VSL#3 in adults with IBS before its use in a randomized study in children with IBS. The researchers used this opportunity to investigate adult patients with IBS with matched fecal omics data and the effects of two dosing durations of VSL#3 on gut permeability, recovery of probiotic organisms, and changes in gut microbiome composition determined by shotgun metagenomic sequencing, as well as alterations in metabolome profile (bile acid). The results of the RCT suggested that the probiotic organisms identified in the stool were significantly correlated with improved colonic permeability and clinical symptoms.

The third article, *Is There a Neuropathic-Like Component to Endometriosis-Associated Pain? Results From a Large Cohort Questionnaire Study* by Coxon et al., reported that clinical data indicate that endometriosis-associated pain includes a neuropathic-like component in a substantial proportion of females. Although further investigation is required, these findings challenge the current conceptualization of endometriosis-associated pain as nociceptive, and the authors advocate for a new perspective on this type of pain. Even within gynecology, related visceral pain probably also results mainly from the neuropathic-like factor (5, 6).

The fourth article, wonderfully related to the psychological gut brain axis, is by Sayuk et al., entitled *Episodic Memories Among Irritable Bowel Syndrome Patients: An Important Aspect of the IBS Symptom Experience*. They identified that 14/29 (48.3%) IBS subjects reported episodic memories of the onset of IBS symptoms, often GI infections/enteritis (35.7%). Episodic memories were associated with greater IBS symptom severity/bother, higher anxiety/depression, and poorer health-related quality of life (HRQOL). However, the subtest memory specificity was not different based on episodic memory. Patients with IBS often reported episodic memory associated with the onset of symptoms, and this recall appears to be associated with more severe symptoms. To date, the much discussed concept of intestinal microflora has been described for the special scientific mechanism; more recently, the main flow mechanism of the nervous and mental axis plays a substantially key role (1). In fact, the nature of abnormal nervous and mental activities should inspire many researchers to make concerted efforts in the treatment of abdominal and pelvic pain and the related interactions.
